# Author Correction: mRNA vaccine developed for sequential selective organ-to-cell targeting of glioma

**DOI:** 10.1038/s41467-026-75443-5

**Published:** 2026-07-09

**Authors:** Lin Shi, Yueying Li, Shiyun Huang, Jin Peng, Chuyao Liu, Zuoyu Hu, Wenbin Deng, Guanjun Deng

**Affiliations:** School of Pharmaceutical Sciences (Shenzhen), Shenzhen Campus of Sun Yat-sen University, Shenzhen, PR China

Correction to: *Nature Communications* 10.1038/s41467-025-67331-1, published online 11 December 2025

In the version of this article initially published, there were errors in the Source Data. For Fig. 2 Source Data, panel d data were inadvertent duplicates of panel e. For Fig. 4 Source Data, hydrodynamic diameters of two nanoparticles in Fig. 4b were inadvertently duplicated; Fig. 4b is now replaced. Figure 5d Source Data had data entry errors for mouse death time points in the PBS and Free mIDH1 groups; the figure has now been replaced. For Supplementary Figs. 15c,d and 16c,d there were labelling errors in the Source Data. Figures 4 and 5 and the Source Data have now been replaced. For comparison, the original Figs. 4 and 5 are available as Supplementary Information accompanying this amendment.

## Supplementary information

Original Figs. 4 and 5

