# Supplementary material for: mRNA vaccine developed for sequential selective organ-to-cell targeting of glioma
Source: Nat Commun. 2025 Dec 11;17:687. doi: 10.1038/s41467-025-67331-1 (PMC12820257; doi:10.1038/s41467-025-67331-1)
Supplement: Supplementary file 2 — Reporting Summary [file 41467_2025_67331_MOESM2_ESM.pdf]

Corresponding author(s): Guanjun Deng and Wenbin Deng

Last updated by author(s): Nov 26, 2025

Reporting Summary

Nature Portfolio wishes to improve the reproducibility of the work that we publish. This form provides structure for consistency and transparency in reporting. For further information on Nature Portfolio policies, see our Editorial Policies and the Editorial Policy Checklist.

Statistics

For all statistical analyses, confirm that the following items are present in the figure legend, table legend, main text, or Methods section.

n/a	Confirmed	
☐	☒	The exact sample size (n) for each experimental group/condition, given as a discrete number and unit of measurement
☐	☒	A statement on whether measurements were taken from distinct samples or whether the same sample was measured repeatedly
☐	☒	The statistical test(s) used AND whether they are one- or two-sided<br>*Only common tests should be described solely by name; describe more complex techniques in the Methods section.*
☒	☐	A description of all covariates tested
☒	☐	A description of any assumptions or corrections, such as tests of normality and adjustment for multiple comparisons
☐	☒	A full description of the statistical parameters including central tendency (e.g. means) or other basic estimates (e.g. regression coefficient) AND variation (e.g. standard deviation) or associated estimates of uncertainty (e.g. confidence intervals)
☐	☒	For null hypothesis testing, the test statistic (e.g. F, t, r) with confidence intervals, effect sizes, degrees of freedom and P value noted<br>*Give P values as exact values whenever suitable.*
☒	☐	For Bayesian analysis, information on the choice of priors and Markov chain Monte Carlo settings
☒	☐	For hierarchical and complex designs, identification of the appropriate level for tests and full reporting of outcomes
☒	☐	Estimates of effect sizes (e.g. Cohen's d, Pearson's r), indicating how they were calculated

Our web collection on statistics for biologists contains articles on many of the points above.

Software and code

Policy information about availability of computer code

Data collection	Zetasizer Pro (Dynamic Light Scattering (DLS) and particle sizing), CytoFLEX (flow cytometer), BD/LSRForte ssa (flow cytometer), OLYMPUS FV3000 (cellular fluorescence imaging system), Tecan/Infinite E plex (for detecting Relative Light Unit (RLU) values of different Lipid Nanoparticles (LNP) after transfection of HEK293T cells), AVANCE NEO Ascend 500 (for identifying chemical structures), PerkinElmer IVIS Lumina III (for in vivo and tumor tissue fluorescence imaging), Case Viewer 2.4 (immunofluorescence image viewer), UVitec Essential V6 (for assessing encapsulation efficiency of Lipid Nanoparticles (LNP)), BG-GDAAUTO730 (Western Blot protein blotting system), Chemdraw 21.0.0 (chemical structure drawing software), Digital Brain Stereotaxer SA301.3 (Tumor Model Construction)
Data analysis	GraphPad Prism 9.5 (Tool for creating heatmaps and bar charts), Origin 2021 (Software for plotting pKa curves), ImageJ (Software for semi-quantitative analysis of fluorescent images), Living Image 4.4 (Software for semi-quantitative analysis of fluorescence in vivo and in tumor tissue), FlowJo_V10 (Software for flow cytometry data analysis), OLYMPUS FLUOVIEW FV31S-SW (OLYMPUS fluorescent image acquisition system), Mestrenova (Software for nuclear magnetic resonance hydrogen spectrum analysis)

For manuscripts utilizing custom algorithms or software that are central to the research but not yet described in published literature, software must be made available to editors and reviewers. We strongly encourage code deposition in a community repository (e.g. GitHub). See the Nature Portfolio guidelines for submitting code & software for further information.

Data

Policy information about availability of data

All manuscripts must include a data availability statement. This statement should provide the following information, where applicable:

- Accession codes, unique identifiers, or web links for publicly available datasets
- A description of any restrictions on data availability
- For clinical datasets or third party data, please ensure that the statement adheres to our policy

The authors declare that the data supporting the findings of this study are available within the paper and its Supplementary Information files. Should any raw data files be needed in another format they are available from the corresponding author upon reasonable request. Source data are provided with this paper.

Research involving human participants, their data, or biological material

Policy information about studies with human participants or human data. See also policy information about sex, gender (identity/presentation), and sexual orientation and race, ethnicity and racism.

Reporting on sex and gender	-
Reporting on race, ethnicity, or other socially relevant groupings	-
Population characteristics	-
Recruitment	-
Ethics oversight	-

Note that full information on the approval of the study protocol must also be provided in the manuscript.

Field-specific reporting

Please select the one below that is the best fit for your research. If you are not sure, read the appropriate sections before making your selection.

☒ Life sciences ☐ Behavioural & social sciences ☐ Ecological, evolutionary & environmental sciences

For a reference copy of the document with all sections, see nature.com/documents/nr-reporting-summary-flat.pdf

Life sciences study design

All studies must disclose on these points even when the disclosure is negative.

Sample size	No sample sizes were predetermined. Sample sizes were at least 3 animals per group. In vitro cell studies included at least 3 replicates per experiment.
Data exclusions	None
Replication	The in vitro experiments were replicated at least three times independently. All animal experiments were replicated at least three times independently.
Randomization	In all studies, samples and animals were assigned randomly to treatment groups.
Blinding	None of the experiments were conducted in a blinded manner.

Reporting for specific materials, systems and methods

We require information from authors about some types of materials, experimental systems and methods used in many studies. Here, indicate whether each material, system or method listed is relevant to your study. If you are not sure if a list item applies to your research, read the appropriate section before selecting a response.

Materials & experimental systems

n/a	Involved in the study	
	x	Antibodies
	x	Eukaryotic cell lines
x		Palaeontology and archaeology
	x	Animals and other organisms
x		Clinical data
x		Dual use research of concern
x		Plants

Methods

n/a	Involved in the study	
x		ChIP-seq
	x	Flow cytometry
x		MRI-based neuroimaging

Antibodies

Antibodies used

DYKDDDDK tag Polyclonal antibody (Binds to FLAG® tag epitope) (Proteintech, Cat#: 20543-1-AP) dilution:1:5000, WB
GAPDH Monoclonal antibody (Proteintech, Cat#: 60004-1-Ig) dilution:1:5000, WB
Goat anti-Rabbit IgG Secondary Antibody HRP conjugated (SAB, Cat#: L3012)
dilution: 1:10000, WB
Brilliant Violet 421™ anti-mouse/human CD11b (Biolegend, clone: M1/70, Cat#: 101235), 1 μL/test, flow cytometry.
PerCP/Cyanine5.5 anti-mouse Siglec H (Biolegend, clone:551, Cat#: 129613), 1 μL/test, flow cytometry.
APC/Fire™ 750 anti-mouse CD8a (Biolegend, clone: 53-6.7, Cat#: 100765), 1 μL/test, flow cytometry.
FITC anti-mouse CD11c (Biolegend, clone: N418, Cat#: 117306), 1 μL/test, flow cytometry.
APC anti-mouse F4/80 (Biolegend, clone: BM8, Cat#: 123115), 1 μL/test, flow cytometry.
FITC anti-mouse CD3 (Biolegend, clone: 500A2, Cat#: 152304), 1 μL/test, flow cytometry.
APC anti-mouse CD4 (Biolegend, clone: GK1.5, Cat#: 100412), 1 μL/test, flow cytometry.
PE anti-mouse IFN-γ (Biolegend, clone: XMG1.2, Cat#: 505868), 1 μL/test, flow cytometry.
Brilliant Violet 421™ anti-mouse IL-4 (Biolegend, clone: 11B11, Cat#: 504119), 1 μL/test, flow cytometry.
FITC anti-mouse NK1.1 (Biolegend, clone: PK136, Cat#: 108705), 1 μL/test, flow cytometry.
PE anti-mouse CD19 (Biolegend, clone: 6D5, Cat#: 115507), 1 μL/test, flow cytometry.
APC anti-mouse CD80 (Biolegend, clone: 16-10A1, Cat#: 104713), 1 μL/test, flow cytometry.
PE anti-mouse CD86 (Biolegend, clone: A17199A, Cat#: 159203), 1 μL/test, flow cytometry.

Validation

Each commercial antibody has been validated for species, application and specificity, as indicated by the manufacturer's website and relevant citations listed by the manufacturer.
Each commercial antibody has been validated for species, application and specificity, as indicated by the manufacturer's website and relevant citations listed by the manufacturer.
DYKDDDDK tag Polyclonal antibody (Binds to FLAG® tag epitope) (Proteintech, Cat#: 20543-1-AP) was validated by successfully staining analysis according to the manufacturer's website https://www.ptgcn.com/products/Flag-Tag-Antibody-20543-1-AP.htm

GAPDH Monoclonal antibody (Proteintech, Cat#: 60004-1-Ig) was validated by successful flow cytometry analysis according to the manufacturer's website https://www.ptgcn.com/products/GAPDH-Antibody-60004-1-Ig.htm

Goat anti-Rabbit IgG Secondary Antibody HRP conjugated (SAB, Cat#: L3012) was validated by successful flow cytometry analysis according to the manufacturer's website https://www.sabbiotech.cn/search.html?cat_name=&keywords=Goat+anti-Rabbit+IgG+Secondary+Antibody+HRP+conjugated+
Brilliant Violet 421™ anti-mouse/human CD11b (Biolegend, clone: M1/70, Cat#: 101235) was validated by successfully staining analysis according to the manufacturer's website https://www.biolegend.com/en-us/products/brilliant-violet-421-anti-mouse-human-cd11b-antibody-7163
PerCP/Cyanine5.5 anti-mouse Siglec H (Biolegend, clone:551, Cat#: 129613) was validated by successfully staining analysis according to the manufacturer's website https://www.biolegend.com/en-us/products/percp-cyanine5-5-anti-mouse-siglec-h-antibody-6927
APC/Fire™ 750 anti-mouse CD8a (Biolegend, clone: 53-6.7, Cat#: 100765) was validated by successfully staining analysis according to the manufacturer's website https://www.biolegend.com/en-us/products/apc-fire-750-anti-mouse-cd8a-antibody-13048
FITC anti-mouse CD11c (Biolegend, clone: N418, Cat#: 117306) was validated by successfully staining analysis according to the manufacturer's website https://www.biolegend.com/en-us/products/fitc-anti-mouse-cd11c-antibody-1815
APC anti-mouse F4/80 (Biolegend, clone: BM8, Cat#: 123115) was validated by successfully staining analysis according to the manufacturer's website https://www.biolegend.com/en-us/products/apc-anti-mouse-f4-80-antibody-4071
FITC anti-mouse CD3 (Biolegend, clone: 500A2, Cat#: 152304) was validated by successfully staining analysis according to the manufacturer's website https://www.biolegend.com/en-us/products/fitc-anti-mouse-cd3epsilon-antibody-13685
APC anti-mouse CD4 (Biolegend, clone: GK1.5, Cat#: 100412) was validated by successfully staining analysis according to the manufacturer's website https://www.biolegend.com/en-us/products/apc-anti-mouse-cd4-antibody-245
PE anti-mouse IFN-γ (Biolegend, clone: XMG1.2, Cat#: 505868) was validated by successfully staining analysis according to the manufacturer's website https://www.biolegend.com/en-us/products/pe-anti-mouse-ifn-gamma-antibody-997
Brilliant Violet 421™ anti-mouse IL-4 (Biolegend, clone: 11B11, Cat#: 504119) was validated by successfully staining analysis according to the manufacturer's website https://www.biolegend.com/en-us/products/brilliant-violet-421-anti-mouse-il-4-antibody-7306
FITC anti-mouse NK1.1 (Biolegend, clone: PK136, Cat#: 108705) was validated by successfully staining analysis according to the manufacturer's website https://www.biolegend.com/en-us/products/fitc-anti-mouse-nk-1-1-antibody-429
PE anti-mouse CD19 (Biolegend ,clone: 6D5, Cat#: 115507) was validated by successfully staining analysis according to the

manufacturer's website https://www.biolegend.com/en-us/products/pe-anti-mouse-cd19-antibody-1530
APC anti-mouse CD80 (Biolegend, clone: 16-10A1, Cat#: 104713) was validated by successfully staining analysis according to the manufacturer's website https://www.biolegend.com/en-us/products/apc-anti-mouse-cd80-antibody-2340
PE anti-mouse CD86 (Biolegend, clone: A17199A, Cat#: 159203) was validated by successfully staining analysis according to the manufacturer's website https://www.biolegend.com/en-us/products/pe-anti-mouse-cd86-antibody-18945

Eukaryotic cell lines

Policy information about cell lines and Sex and Gender in Research

Cell line source(s)	HEK293T, DC2.4, GL261
Authentication	The cell lines were authenticated by short tandem repeat (STR) analysis.
Mycoplasma contamination	The cell lines were tested for mycoplasma contamination via loop-mediated isothermal amplification (LAMP) to ensure the absence of mycoplasma.
Commonly misidentified lines (See ICLAC register)	No commonly misidentified cell lines were used.

Animals and other research organisms

Policy information about studies involving animals; ARRIVE guidelines recommended for reporting animal research, and Sex and Gender in Research

Laboratory animals	Male BALB/c mice (6–8 weeks old, 20g) and male C57 mice (6–8 weeks old, 20g) were bred in a temperature- and humidity-controlled SPF condition (20-25°C and 45-55% humidity) under 12/12h light/dark cycles with free access to food and water. The maximum tumor volume/load approved by the Sun Yat-sen University Ethics Committee is that tumor growth must not exceed 10 % of the animal's original body weight and that the average tumor diameter in mice must not exceed 20 mm; we confirm that these limits were not exceeded during the experiment. All animal husbandry and experimental procedures were approved by the Animal Ethics Committee of the Laboratory Animal Research Center of Sun Yat-sen University.
Wild animals	The study did not involve wild animals.
Reporting on sex	No sex-based stratification or sex-based analyses were performed.
Field-collected samples	The study did not involve samples collected from the field.
Ethics oversight	All animals were handled in accordance with the policies and guidelines of the Animal Ethics Committee of the Laboratory Animal Research Center of Sun Yat-sen University (Animal Ethics Committee approval number: AEDGJ202301)

Note that full information on the approval of the study protocol must also be provided in the manuscript.

Plants

Seed stocks	-
Novel plant genotypes	-
Authentication	-

Flow Cytometry

Plots

Confirm that:

- [ ] The axis labels state the marker and fluorochrome used (e.g. CD4-FITC).
- [x] The axis scales are clearly visible. Include numbers along axes only for bottom left plot of group (a 'group' is an analysis of identical markers).
- [x] All plots are contour plots with outliers or pseudocolor plots.
- [x] A numerical value for number of cells or percentage (with statistics) is provided.

Methodology

Sample preparation

Ai9 mice were intravenously injected with the prepared mXO10 tLNP@Cre mRNA at a dose of 0.2 mg/kg and 0.5 mg/kg. After 48 h, the spleen was harvested, minced, and passed through a 70 µm filter to create a cell suspension. The cell suspension was then lysed with red blood cell lysis buffer for 3 min on ice, followed by Incubating with CD16/32 for 10 minutes.. Different types of immune cells were labeled using specific antibodies: DC cells were labeled with CD11c antibody, macrophages were labeled with CD11b and F4/80 antibodies, NK cells were labeled with NK1.1 antibody, B cells were labeled with CD19 antibody, and T cells were labeled with CD3 antibody.

Instrument

CytoFLEX

Software

FlowJo_V10

Cell population abundance

No cell sorting was performed in this study.

Gating strategy

First, cells are gated based on their size (using FSC-A and SSC-A) to distinguish different cell populations. Next, adherent cells are removed by comparing FSC-H and FSC-A. Then, tdtomato-positive cells are identified using the PE filter. Among the tdtomato-positive cells, further differentiation is made to identify specific cell subtypes: DC cells are identified using the APC filter, NK cells using the FITC filter, macrophages using both FITC and APC filters, B cells using the PE-Cy7 filter, and T cells using the APC filter

☐ Tick this box to confirm that a figure exemplifying the gating strategy is provided in the Supplementary Information.
