# Supplementary material for: mRNA vaccine developed for sequential selective organ-to-cell targeting of glioma
Source: Nat Commun. 2025 Dec 11;17:687. doi: 10.1038/s41467-025-67331-1 (PMC12820257; doi:10.1038/s41467-025-67331-1)
Supplement: Supplementary file 2 [file 41467_2025_67331_MOESM1_ESM.pdf]

Supplementary Information

mRNA Vaccine developed for Sequential Selective Organ-to-Cell Targeting of Glioma

Lin Shi [1, ‡], Yueying Li [1, ‡], Shiyun Huang [1], Jin Peng [1], Chuyao Liu [1], Zuoyu Hu [1], Wenbin Deng [1,*], Guanjun Deng [1,*]

[1] School of Pharmaceutical Sciences (Shenzhen), Shenzhen Campus of Sun Yat-sen University, Shenzhen 518107, P. R. China

[‡] These authors contributed equally to this work.

[*] Correspondence authors: Guanjun Deng (E-mail: denggj3@mail.sysu.edu.cn) and Wenbin Deng (E-mail: dengwb5@mail.sysu.edu.cn)

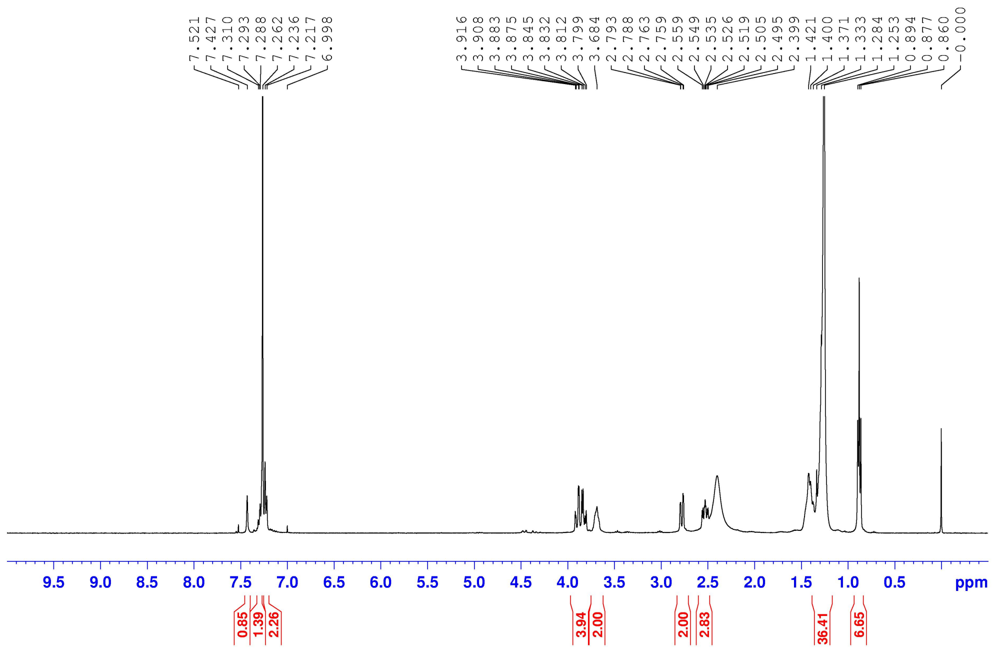

Figure S1. Characterization of lipidoids. ^{1}H-NMR spectrum of mXO10 lipidoids.

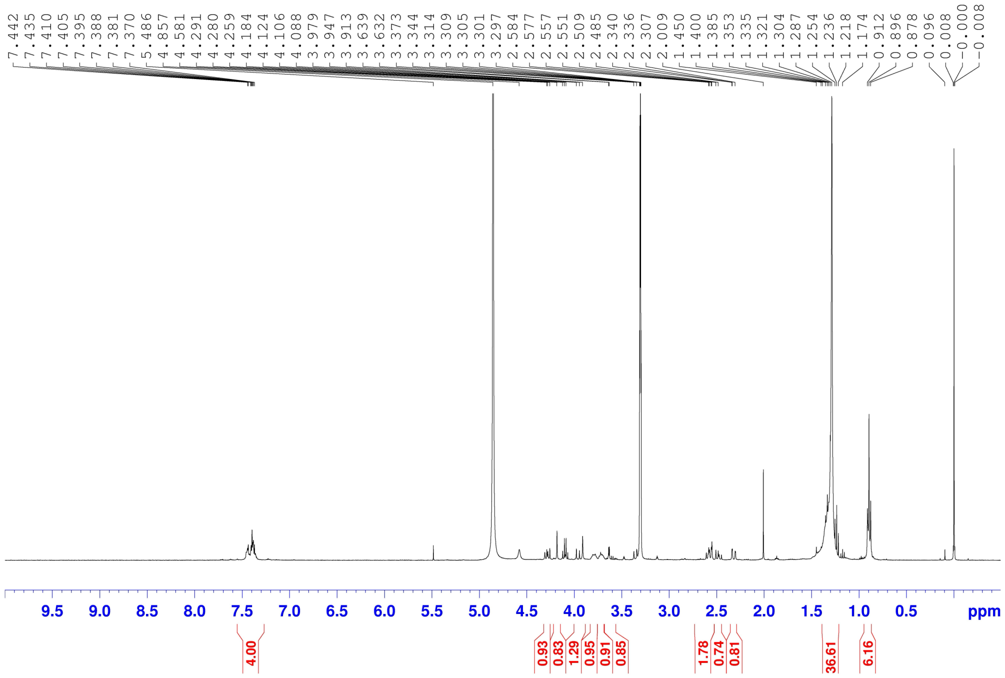

Figure S2. Characterization of lipidoids. ^{1}H-NMR spectrum of oXO10 lipidoids.

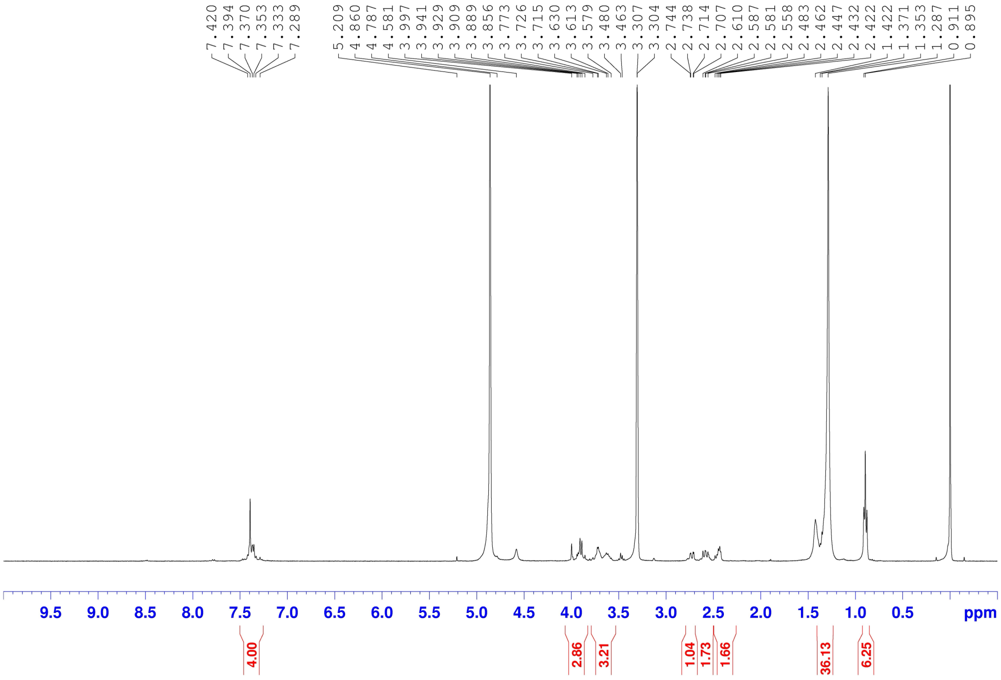

Figure S3. Characterization of lipidoids. ¹H-NMR spectrum of pXO10 lipidoids.

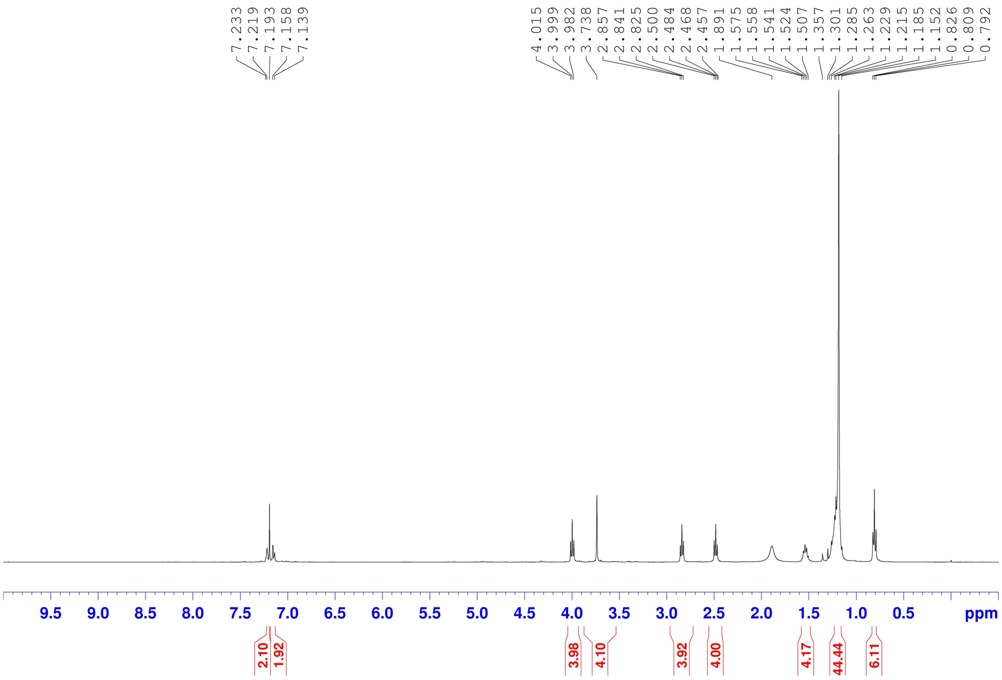

Figure S4. Characterization of lipidoids. ¹H-NMR spectrum of mXE14 lipidoids.

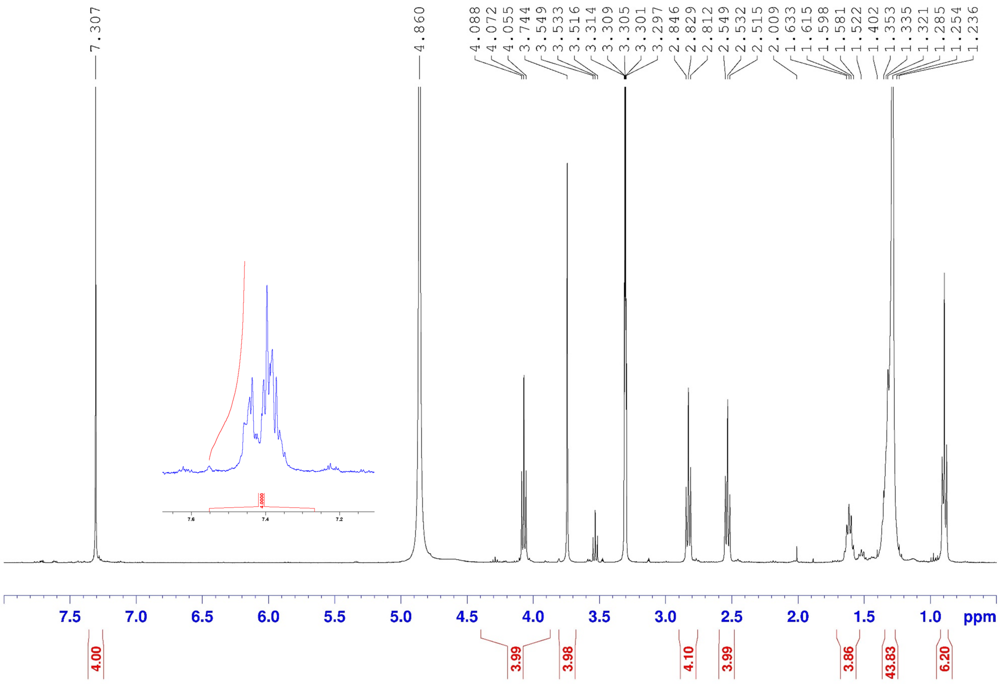

Figure S5. Characterization of lipidoids. ^{1}H-NMR spectrum of oXE14 lipidoids.

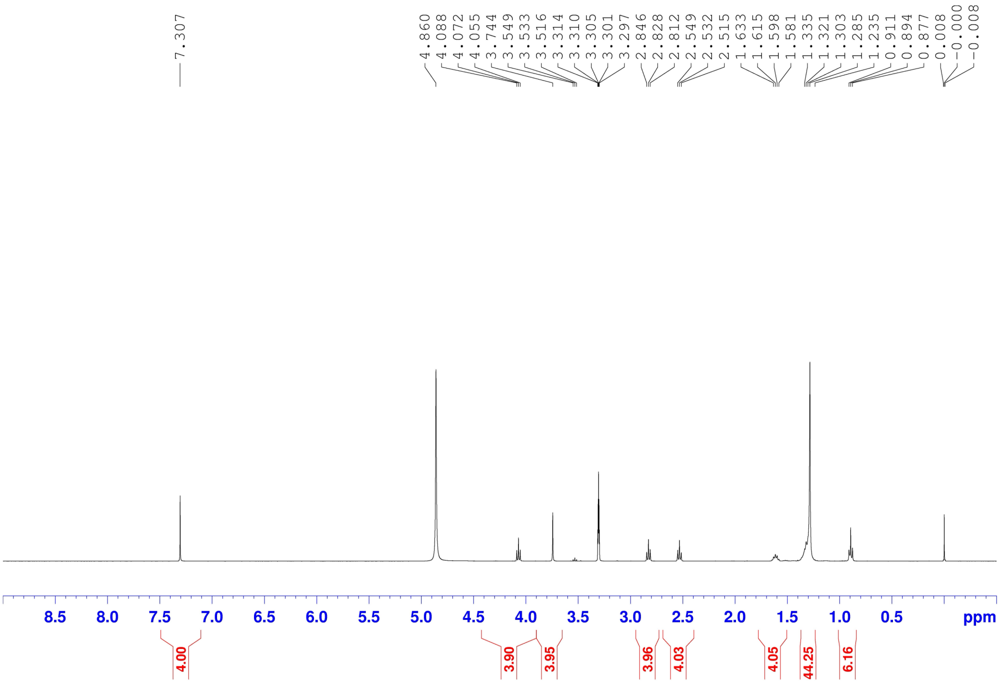

Figure S6. Characterization of lipidoids. ^{1}H-NMR spectrum of pXE14 lipidoids.

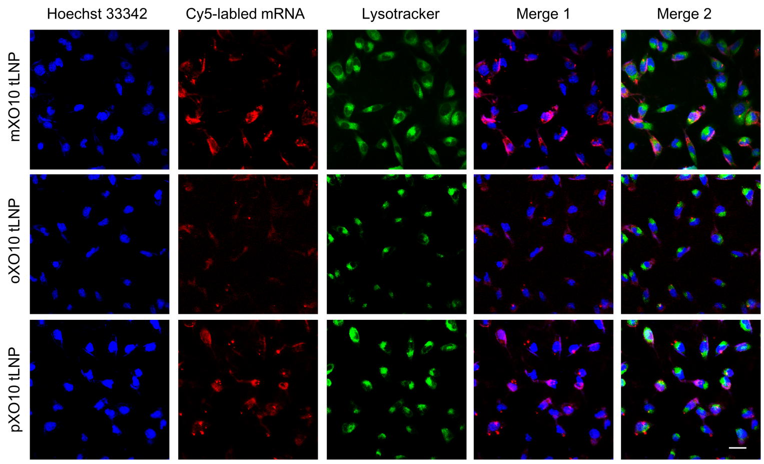

Figure S7. Study on cellular uptake and lysosomal escape of mRNA delivered by tLNP in DC2.4 cells. Representative confocal images showed cellular uptake and endosomal escape of mXO10 tLNP, oXO10 tLNP, and pXO10 tLNP. mRNA was labeled with Cy5 (Red), lysosomes were labeled with Lysotracker (Green), and nuclei were labeled with Hoechst 33342 (Blue). Scale bar 20 µm. (n = 3 independent experiments）

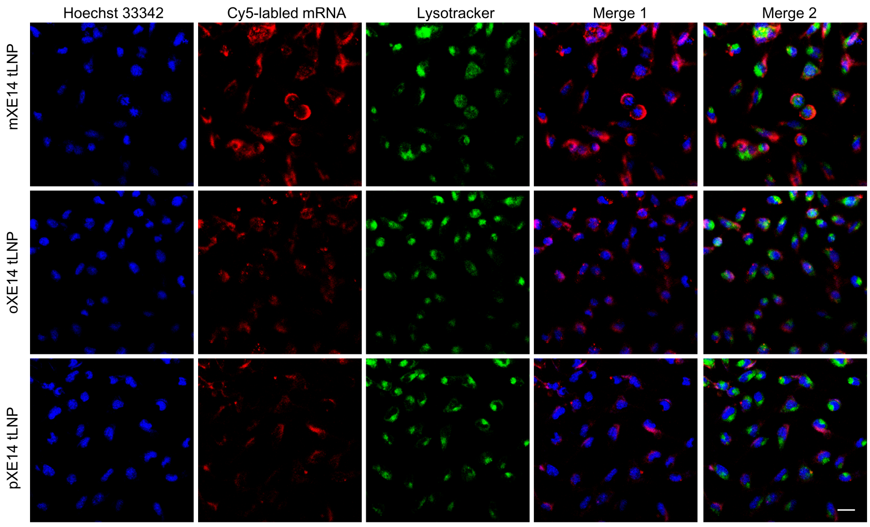

Figure S8. Study on cellular uptake and lysosomal escape of mRNA delivered by tLNP in DC2.4 cells. Representative confocal images showed cellular uptake and endosomal escape of mXE14/oXE14/pXE14 tLNP in DC2.4 cells. mRNA was labeled with Cy5, lysosomes were stained with Lysotracker (green), and nuclei were stained with Hoechst 33342 (blue). Scale bar 20 µm. (n = 3 independent experiments）

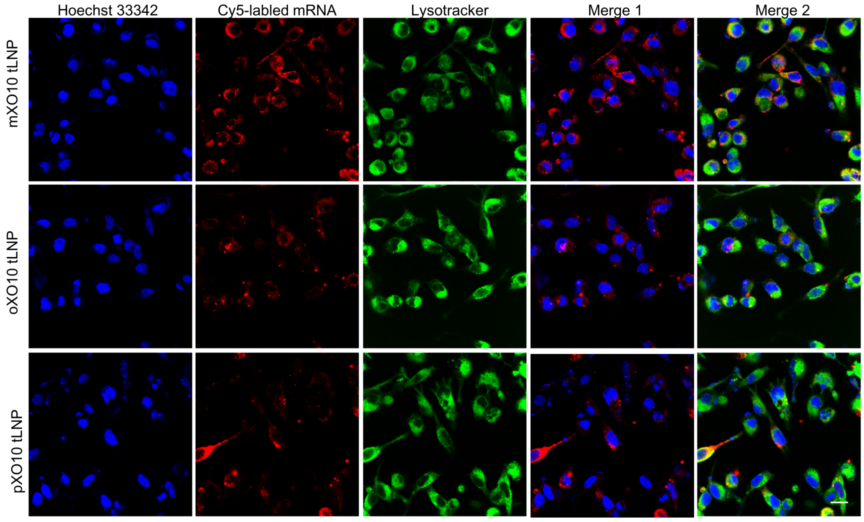

Figure S9. Study on cellular uptake and lysosomal escape of mRNA delivered by mXO10/oXO10/pXO10 tLNP in GL261 cells. Representative confocal images demonstrating cell uptake and endosome escape of mXO10/oXO10/pXO10 tLNP in GL261 cells. Following a 4-hour incubation with Cy5-labeled mRNA encapsulated within the tLNP, the cells were stained with Lysotracker (green) to visualize endosomes and Hoechst 33342 (blue) to stain the nuclei. Scale bar 20 µm. (n = 3 independent experiments）

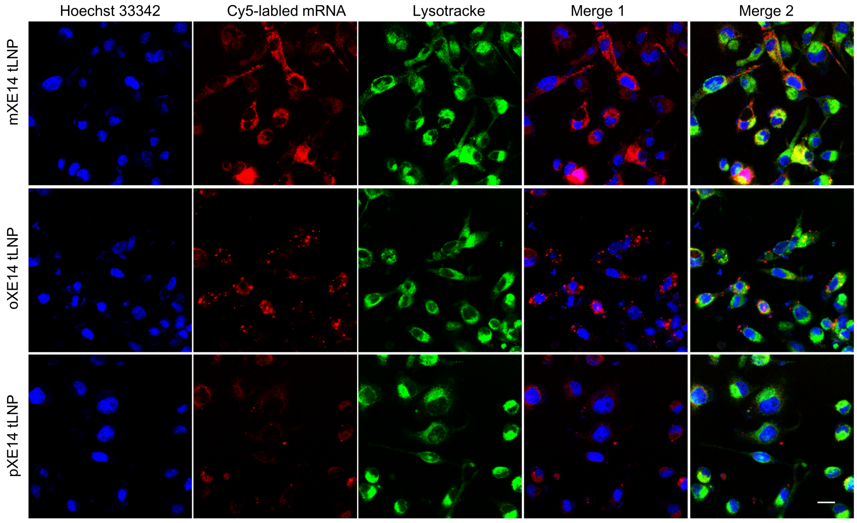

Figure S10. Study on cellular uptake and lysosomal escape of mRNA delivered by mXE14/oXE14/pXE14 tLNP in GL261 cells. Representative confocal images presented cell uptake and LNP endosome escape of mXE14/oXE14/pXE14 tLNPs in GL261 cells. Following a 4-hour incubation with Cy5-labeled mRNA encapsulated within tLNP, the cells were stained with Lysotracker (green) to visualize endosomes and Hoechst 33342 (blue) to stain the nuclei. Scale bar 20 µm. (n = 3 independent experiments）

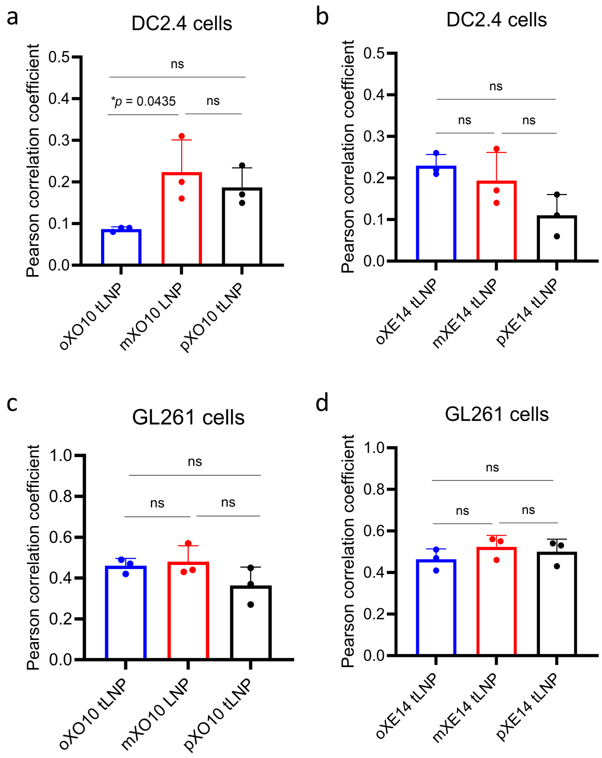

Figure S11. Study on lysosomal escape of mRNA delivered by meta/ortho-/para-tLNP in DC2.4 and GL261 cells. a. Semi-quantitative analysis of lysosomal escape of mXO10/oXO10/pXO10 tLNP in DC2.4 cells (a) and GL261 cells (c). Semi-quantitative analysis of lysosomal escape of mXE14/oXE14/pXE14 tLNP in DC2.4 cells (b) and GL261 cells (d). (n = 3 independent experiments, data were presented as mean ± SEM, and analyzed using one-way ANOVA followed by Tukey post hoc test, 95% confidence interval, $*p < 0.05$, $**p < 0.01$, $***p < 0.001$, $****p < 0.0001$, n.s., not significant.) Source data and exact p values are provided as a Source Data file.

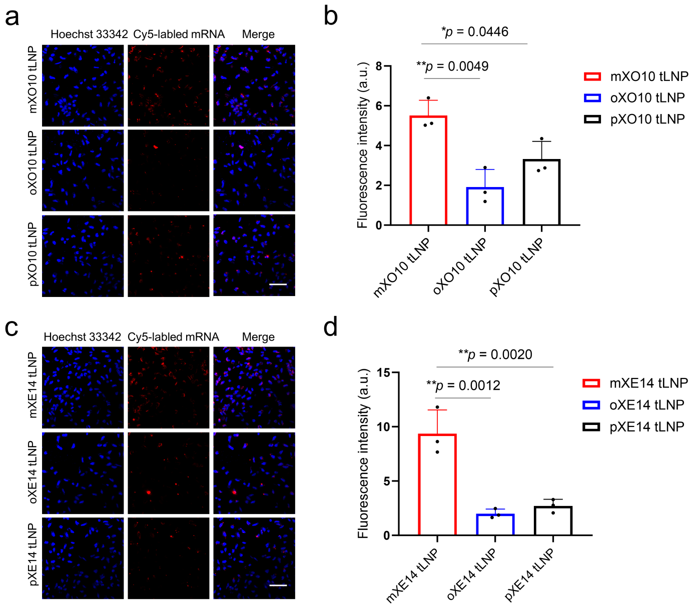

Figure S12. Study on cellular uptake of mRNA delivered by tLNP. a. Representative confocal

images showing the cellular uptake of mXO10/oXO10/pXO10 tLNP in DC2.4 cells. Scale bar 100 µm. (n = 3 independent experiments) b. Semi-quantitative analysis of cellular uptake of mXO10/oXO10/pXO10 tLNP from (a). c. Representative confocal images showing the cellular uptake of mXE14 tLNP, oXE14 tLNP, and pXE14 tLNP in DC2.4 cells. (n = 3 independent experiments) d. Semi-quantitative analysis of cellular uptake of mXE14/oXE14/pXE14 tLNP in DC2.4 cells. (n = 3 independent experiments, data were presented as mean ± SEM, and analyzed using one-way ANOVA followed by Tukey post hoc test, 95% confidence interval, **p* < 0.05, ***p* < 0.01, ****p* < 0.001, *****p* <0.0001, n.s., not significant.) Source data and exact *p* values are provided as a Source Data file.

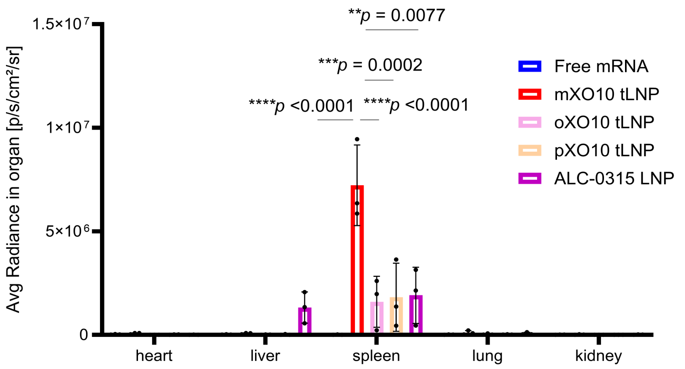

Figure S13. Semi-quantification of *ex vivo* organ images (heart, liver, spleen, lungs, kidneys) in figure 3b. (n = 3 independent experiments) Source data are provided as a Source Data file.

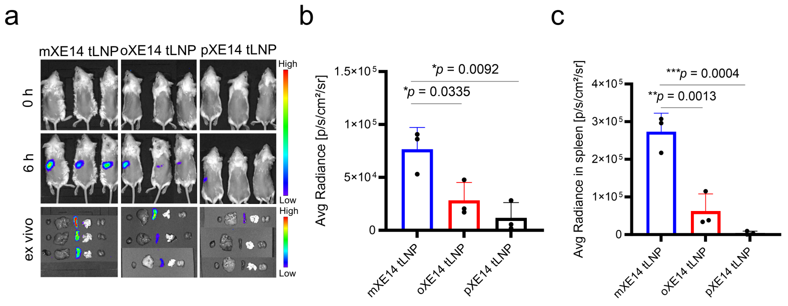

Figure S14. a. Study on *in vivo* delivery of mRNA by mXE14 tLNP, oXE14 tLNP, and pXE14 tLNP. (n = 3 independent experiments) b. Semi-quantitative analysis of *in vivo* spleen at 6 h in figure S14a. c. Semi-quantitative analysis of the *ex vivo* spleen images in figure S14a. (n = 3 independent experiments, data were presented as mean ± SEM, and analyzed using one-way ANOVA followed by Tukey post hoc test, 95% confidence interval, **p* < 0.05, ***p* < 0.01, ****p* < 0.001, *****p* <0.0001, n.s., not significant.) Source data and exact *p* values are provided as a Source Data file.

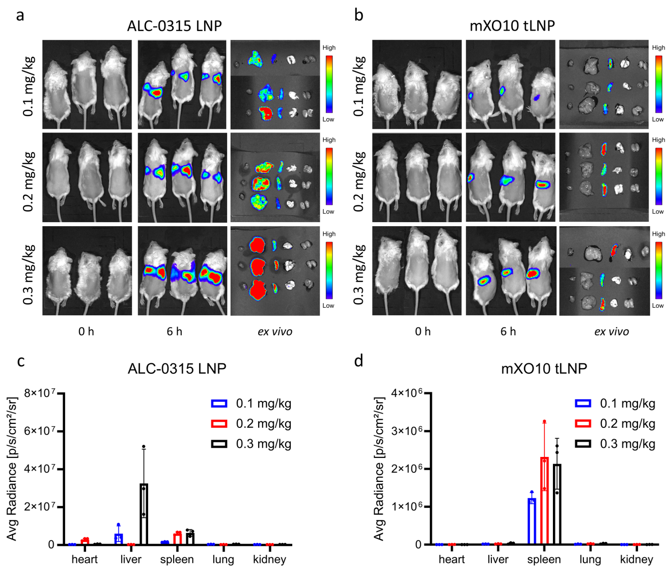

Figure S15. Dose-dependent expression evaluation of ALC-0315 LNP and mXO10 tLNP. a. Expression of ALC-0315 LNP at different doses: expression levels after administration of ALC-0315 LNP at doses of 0.1 mg/kg, 0.2 mg/kg, and 0.3 mg/kg. b. Expression of mXO10 tLNP at different doses: expression levels after administration of mXO10 tLNP at doses of 0.1 mg/kg, 0.2 mg/kg, and 0.3 mg/kg. c and d. Semi-quantitative analysis of the expression levels in a and b, respectively. (n = 3 independent experiments, data were presented as mean ± SEM.) Source data are provided as a Source Data file.

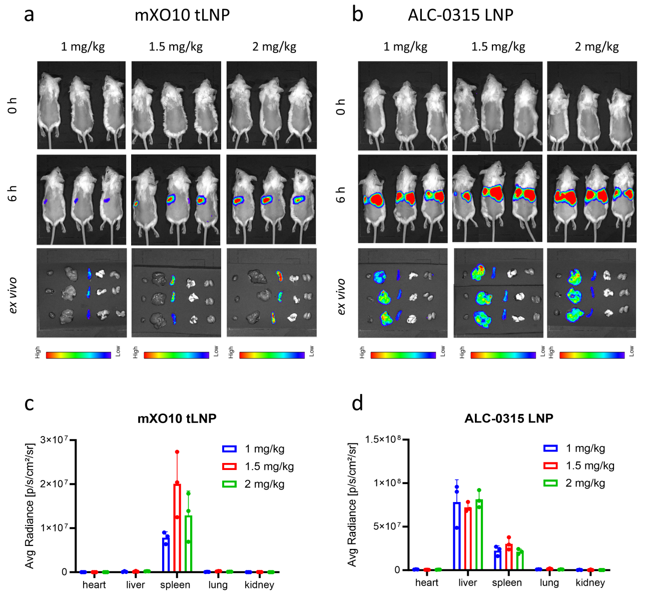

Figure S16. Dose-dependent mRNA expression evaluation of ALC-0315 LNP and mXO10 tLNP. a. mRNA expression of mXO10 tLNP at different doses (mRNA expression levels after administration of mXO10 tLNP at doses of 1 mg/kg, 1.5 mg/kg, and 2 mg/kg). b. mRNA expression of ALC-0315 LNP at different doses (mRNA expression levels after administration of ALC-0315 LNP at doses of 1 mg/kg, 1.5 mg/kg, and 2 mg/kg). c and d. Semi-quantitative analysis of the expression levels in a and b, respectively. (n = 3 independent experiments, data were presented as

mean ± SEM.) Source data are provided as a Source Data file.

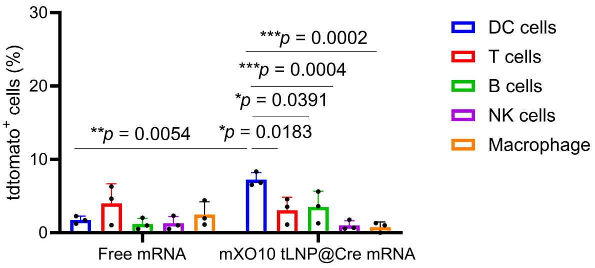

Figure S17. The development of SSOCT. Study on targeted delivery of mRNA to cells in the spleen using mX10 tLNP at a dose of 0.2 mg/kg. (n = 3 independent experiments, data were presented as the mean ± SEM, and analyzed using two-way ANOVA followed by Tukey post hoc test, 95% confidence interval, **p* < 0.05, ***p* < 0.01, ****p* < 0.001, *****p* <0.0001, n.s., not significant.) Source data and exact *p* values are provided as a Source Data file.

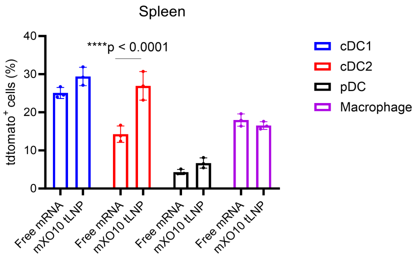

Figure S18. The development of SSOCT. Study on the targeted expression of mRNA by mXO10 tLNP on DC subsets. (n = 3 independent experiments, data are presented as mean ± SEM, and analyzed using two-way ANOVA followed by Tukey post hoc test, 95% confidence interval, **p* < 0.05, ***p* < 0.01, ****p* < 0.001, *****p* <0.0001, n.s., not significant.) Source data and exact *p* values are provided as a Source Data file.

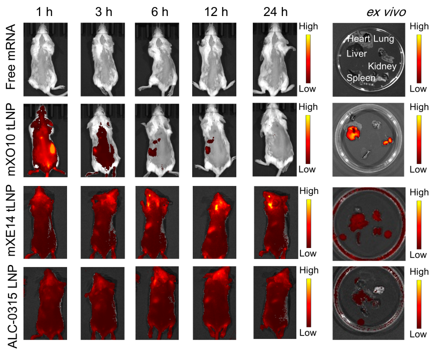

Figure S19. To assess the biodistribution of tLNP in mice by fluorescence imaging, we labeled the mRNA with Cy5 dye.

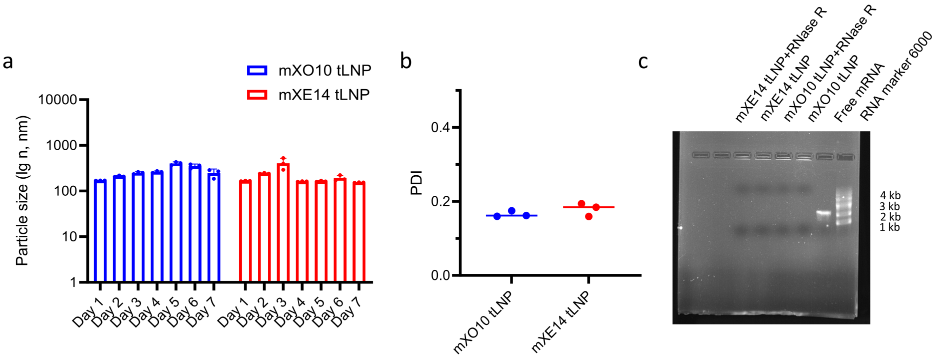

Figure S20. Characterization of mXO10 tLNP and mXE14 tLNP. a and b. Stability study of mXO10 tLNP and mXE14 tLNP. c. Agarose gel electrophoresis to evaluate the resistance of mRNA delivered by mXO10 tLNP (or mXE14 tLNP) to RNase (200 ng/μL) and the study of mRNA encapsulation. (n = 3 independent experiments, data were presented as the mean ± SEM.)

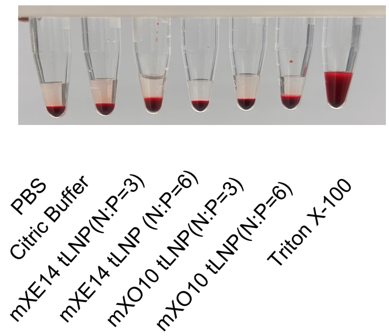

Figure S21. The biocompatibility of tLNP. Hemolysis experiments of mXO10 tLNP (or mXE14 tLNP) with different N:P ratios.

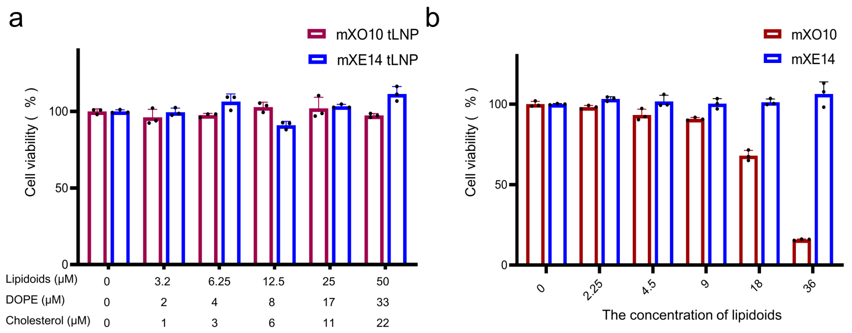

Figure S22. The biocompatibility of tLNP. a. CCK-8 cytotoxicity of mXO10 tLNP and mXE14 tLNP. b. CCK-8 cytotoxicity of lipidoids (mXO10 and mXE14). (n = 3 independent experiments, data were presented as the mean ± SEM.)

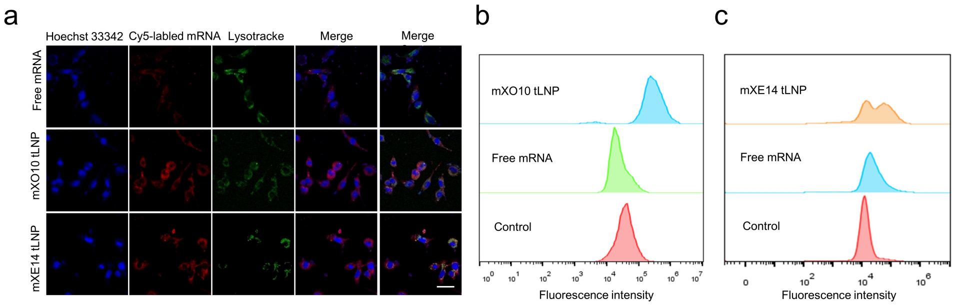

Figure S23. The study cellular uptake and lysosomal escape of mRNA delivered by mXO10 tLNP and mXE14 tLNP in GL261 cells. a. Representative confocal images illustrating cell uptake and lysosomal escape for mXO10 tLNP and mXE14 tLNP in GL261 cells. After a 4-hour incubation with Cy5-labeled mRNA encapsulated within the tLNP, the cells were stained with Lysotracker (green) to visualize Lysosome and Hoechst 33342 (blue) to stain the nuclei. Scale bar 20 µm. (n = 3 independent experiments) The uptake efficiency of mRNA encapsulated within mXO10 tLNP (b) and mXE14 tLNP (c) by GL261 cells was assessed using flow cytometry.

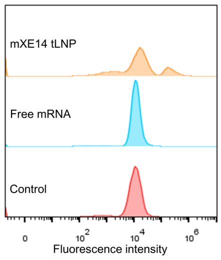

Figure S24. The study cellular uptake of mRNA delivered by mXE14 tLNP in DC2.4 cells. The uptake efficiency of mRNA encapsulated within mXE14 tLNP by DC2.4 cells was evaluated using flow cytometry.

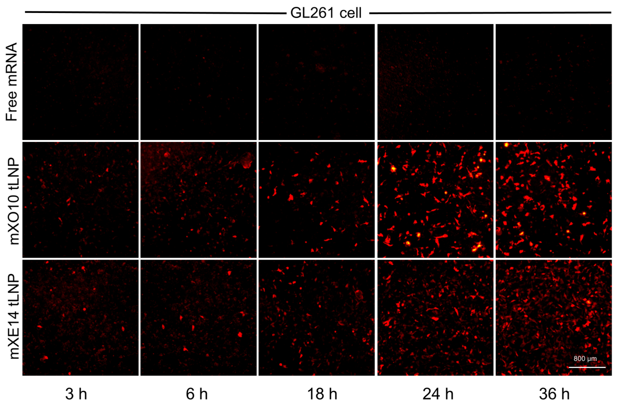

Figure S25. Study of the efficiency of tLNP-mediated mRNA delivery into GL261 cells. Protein expression of mCherry mRNA delivered by mXO10 tLNP and mXE14 tLNP in GL261 cells. (n = 3 independent experiments)

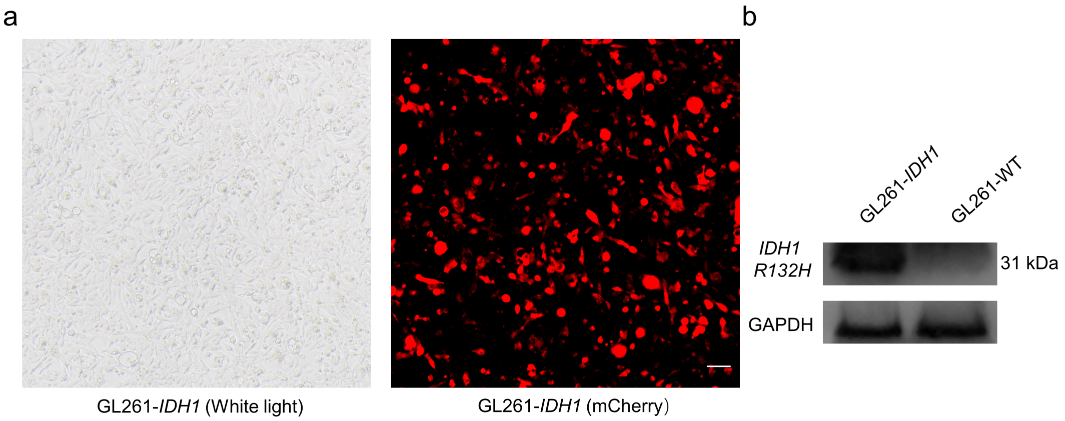

Figure S26. Construction of GL261-*IDH1 R132H* Stable Cell Line. a. Cell morphology and mCherry fluorescence expression: After puromycin selection, the morphology of cells under bright-field microscopy and the expression of mCherry fluorescent protein under fluorescence microscopy were observed. Scale bar 100 µm. b. Western blot validation: Western blot analysis was performed to verify the expression of the IDH1 gene in GL261 cells. (n = 3 independent experiments)

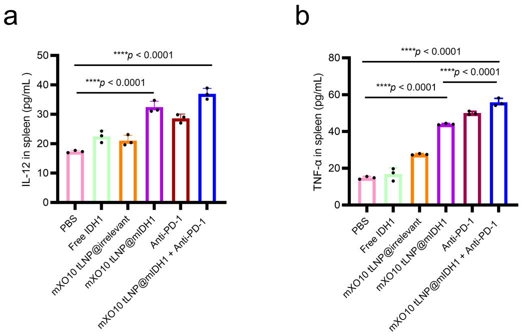

Figure S27. Study on the determination of in vivo cytokines. Measurement of cytokine IL-12 (a) and TNF-α (b) levels in mouse spleens after different treatments using ELISA. (n = 3 independent experiments, data were presented as mean ± SEM, and analyzed using one-way ANOVA followed by Tukey post hoc test, 95% confidence interval., $*p < 0.05$, $**p < 0.01$, $***p < 0.001$, $****p$ <0.0001, n.s., not significant.) Source data and exact p values are provided as a Source Data file.

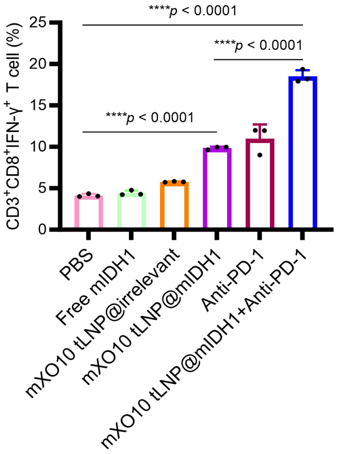

Figure S28. Flow cytometry analysis of the proportion of $CD3^{+}CD8^{+}IFN\text{-}\gamma^{+}$ T cells in the spleen. This analysis was conducted using non-specific stimulation with PMA and ionomycin stimulation. (n = 3 independent experiments, data were presented as mean ± SEM, and analyzed using one-way ANOVA followed by Tukey post hoc test, 95% confidence interval., **p* < 0.05, ***p* < 0.01, ****p* < 0.001, *****p* <0.0001, n.s., not significant.) Source data and exact *p* values are provided as a Source Data file.

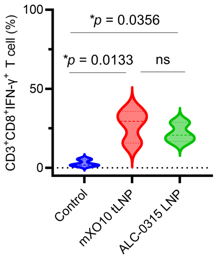

Figure S29. Determination of *IDH1 R132H*-specific $CD3^{+}CD8^{+}IFN\text{-}\gamma^{+}$ T cells in the mXO10 tLNP@mIDH1 group. Flow cytometric statistical analysis of *IDH1 R132H*-specific $CD3^{+}CD8^{+}IFN\text{-}\gamma^{+}$ T cells. (n = 3 independent experiments, data were presented as mean ± SEM, and analyzed using one-way ANOVA followed by Tukey post hoc test, 95% confidence interval., **p* < 0.05, ***p* < 0.01, ****p* < 0.001, *****p* <0.0001, n.s., not significant.) Source data and exact *p* values are provided as a Source Data file.

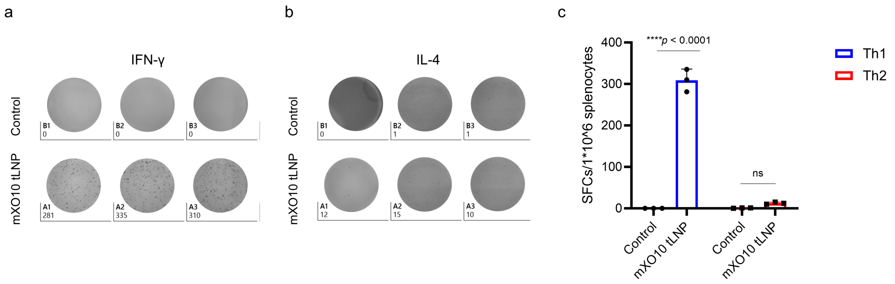

Figure S30. Determination of cytokines IFN-γ and IL-4. a and b. The secretion levels of the cytokines IFN-γ and IL-4 following treatment with mXO10 tLNP were evaluated using the ELISpot assay. c. The secretion of IFN-γ and IL-4 cytokines after treatment with mXO10 tLNP was quantitatively assessed using the ELISpot assay. (n = 3 independent experiments, data are presented as mean ± SEM, and analyzed using two-way ANOVA followed by Tukey post hoc test, 95% confidence interval, $*p < 0.05$, $**p < 0.01$, $***p < 0.001$, $****p < 0.0001$, n.s., not significant.) Source data and exact *p* values provided as a Source Data file.

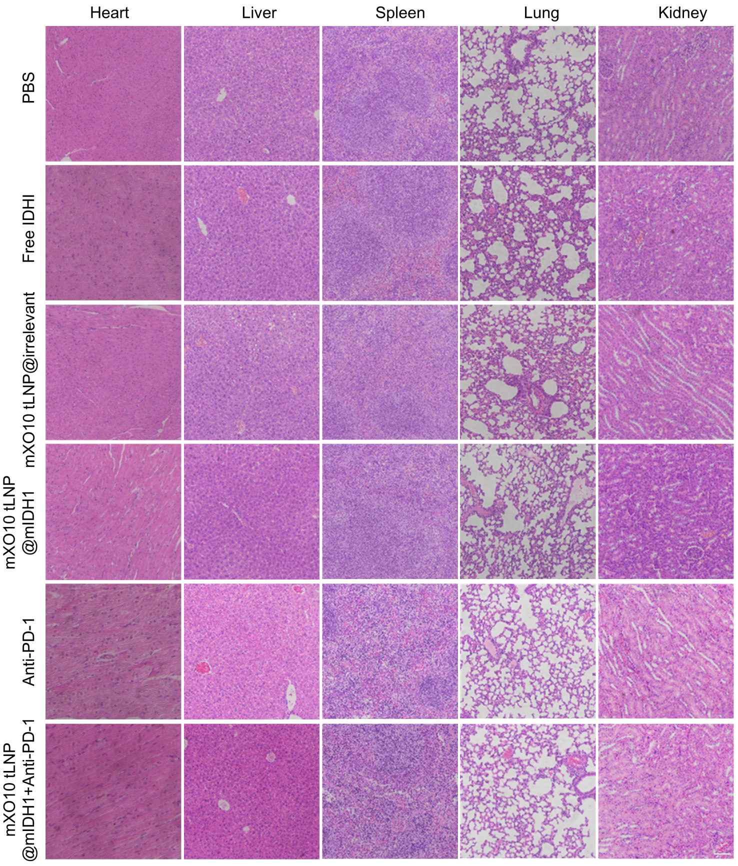

Figure S31. Study on biocompatibility. HE staining of heart, liver, spleen, lung and kidney. Scale bar 100 μm.

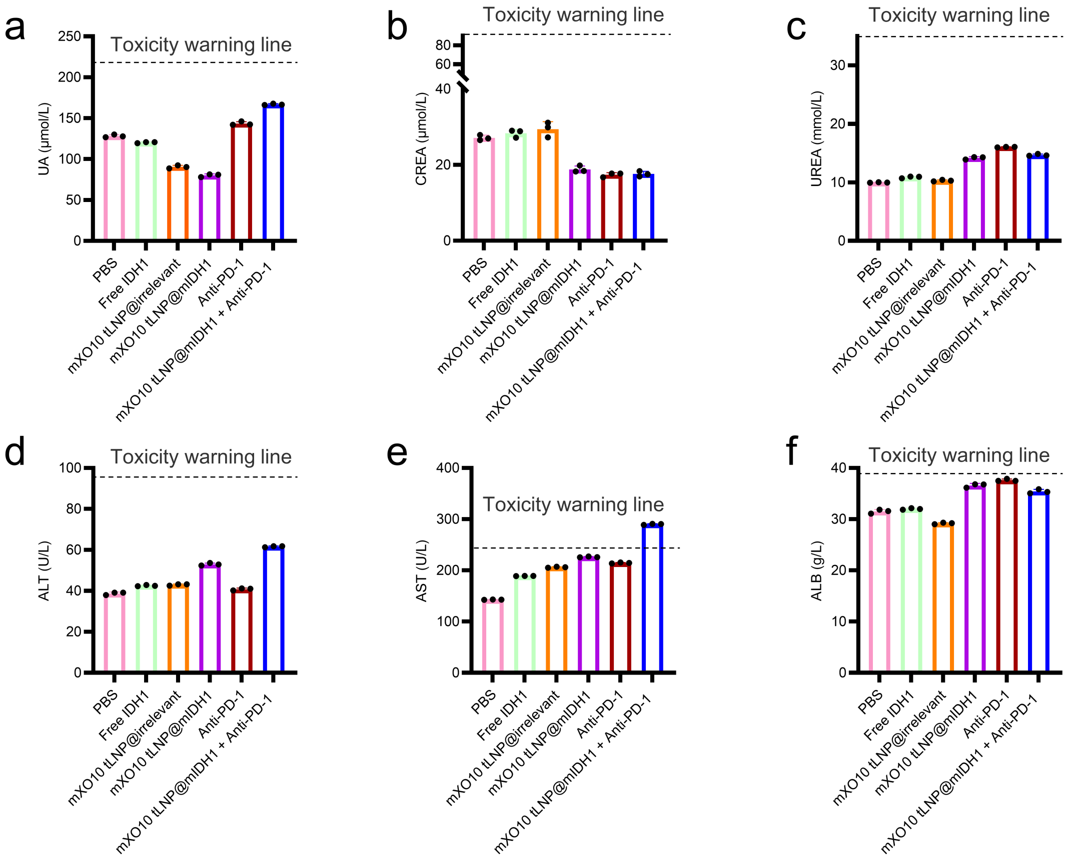

Figure S32. Study on biocompatibility. The measurement of biochemical indexes related to liver and kidney function. (n = 3 independent experiments, data are presented as mean ± SEM.) Source data are provided as a Source Data file.
