# Supplementary material for: Author Correction: mRNA vaccine developed for sequential selective organ-to-cell targeting of glioma
Source: Nat Commun. 2026 Jul 9;17:6046. doi: 10.1038/s41467-026-75443-5 (PMC13350861; doi:10.1038/s41467-026-75443-5)
Supplement: Supplementary file 1 — Original Figs. 4 and 5 [file 41467_2026_75443_MOESM1_ESM.pdf]

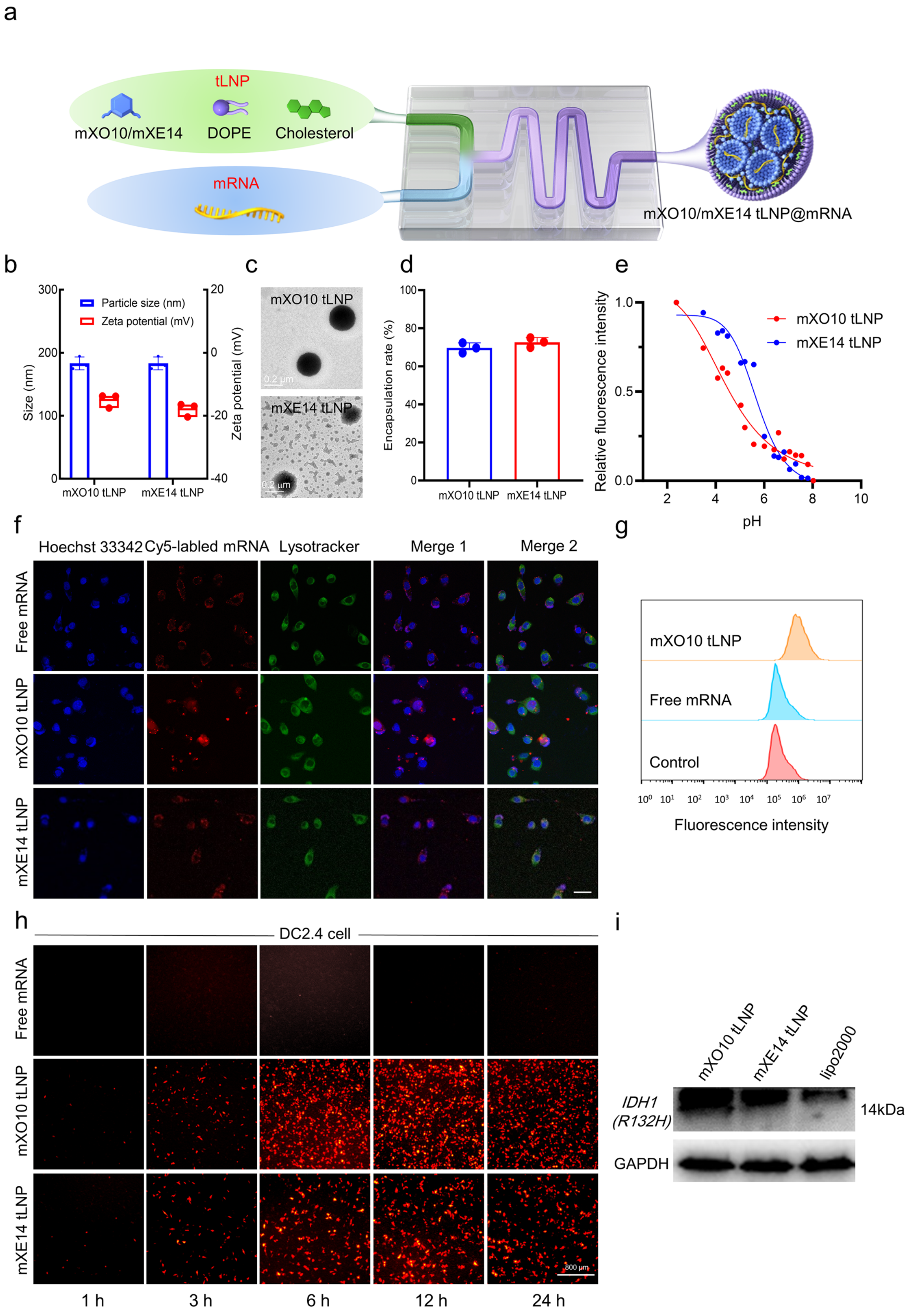

critical factor in mRNA delivery. We studied the lysosomal escape of tLNP using CLSM. The result showed that, compared to the control group, naked mRNA could barely enter DC2.4 cells, whereas mXO10 tLNP@mRNA and mXE14 tLNP@mRNA showed significant lysosomal escape and a substantial amount of mRNA entering the cytoplasm, providing a foundation for efficient protein expression. The result indicated that mXO10 tLNP and mXE14 tLNP significantly improved the efficiency of mRNA uptake and lysosomal escape, enhancing the delivery and translation of mRNA into proteins. Previous data have shown that mXO10 tLNP and mXE14 tLNP effectively deliver luciferase mRNA (mLuc) into cells and efficiently translate it into luciferase. Next, we explored whether these tLNP could deliver other mRNA and efficiently translate them into proteins. mCherry mRNA (mCherry) encodes a red fluorescent protein. We used mXO10 tLNP and mXE14

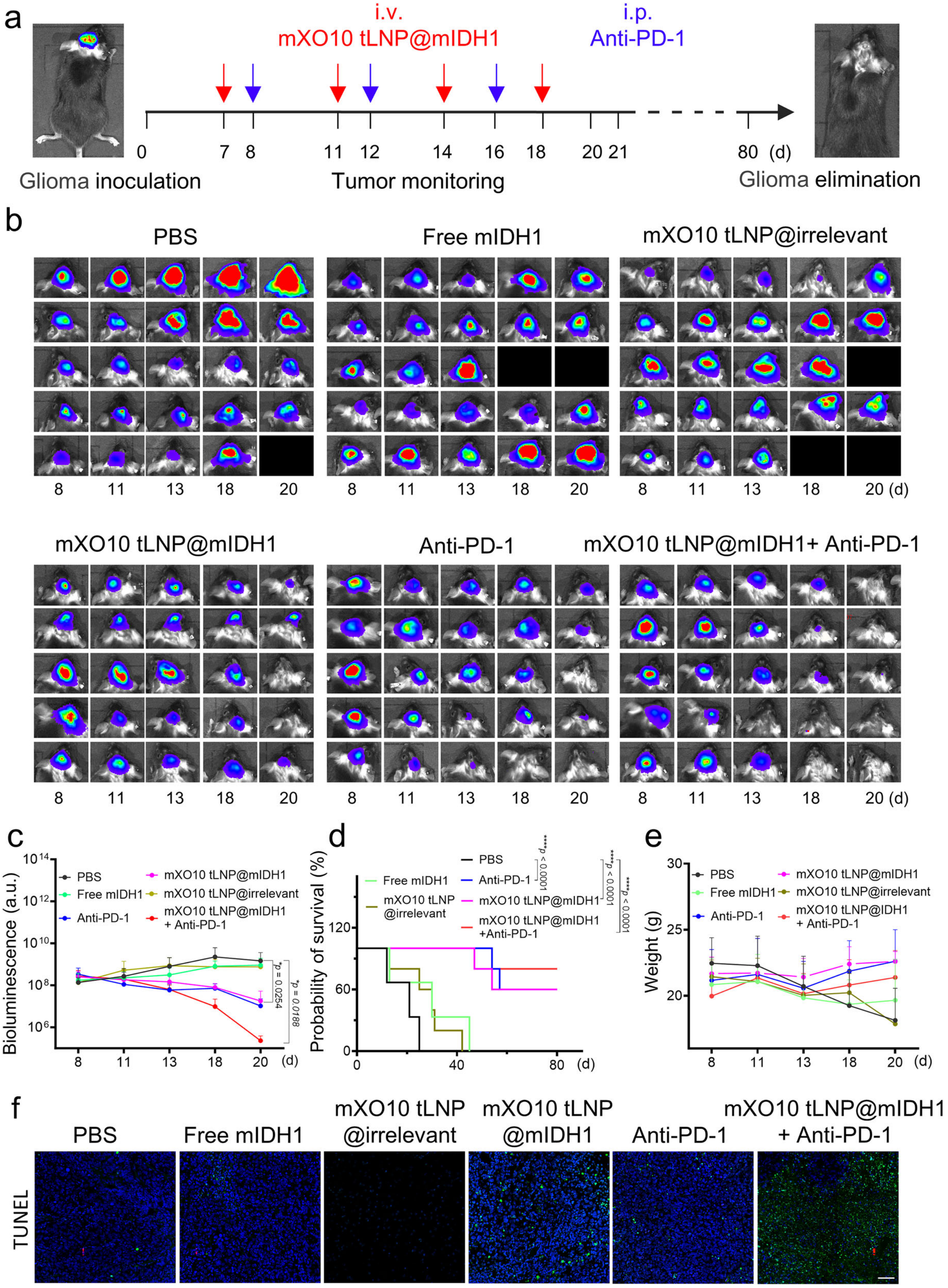

Fig. 5 | Study on the treatment of glioma using mRNA vaccine combined with Anti-PD-1 therapy. a Schematic diagram of the treatment process for glioma using the mXO10 tLNP@mIDH1 vaccine combined with Anti-PD-1. **b** Bioluminescence imaging study to monitor glioma growth in vivo. **c** Semi-quantitative analysis of bioluminescence signals in glioma from (**b**) after different treatments. **d** Study on the survival time of mice after different treatments (PBS vs mXO10 tLNP@mIDH1, ****p <0.0001; PBS vs mXO10 tLNP@mIDH1+Anti-PD-1, ****p <0.0001.). **e** Study on the monitoring of mouse body weight during treatment. (n = 5 independent experiments, data were presented as mean ± SEM) **f** Study on apoptosis of glioma cells in mice after different treatments. (n = 3 independent experiments, data were presented as mean ± SEM.) Scale bar 100 μm. (n = 3 independent experiments) Source data are provided as a Source data file.
